# Psychophysical Evaluation of Congenital Colour Vision Deficiency: Discrimination between Protans and Deutans Using Mollon-Reffin’s Ellipses and the Farnsworth-Munsell 100-Hue Test

**DOI:** 10.1371/journal.pone.0152214

**Published:** 2016-04-21

**Authors:** Natáli Valim Oliver Bento-Torres, Anderson Raiol Rodrigues, Maria Izabel Tentes Côrtes, Daniela Maria de Oliveira Bonci, Dora Fix Ventura, Luiz Carlos de Lima Silveira

**Affiliations:** 1 Instituto de Ciências Biológicas, Universidade Federal do Pará, Belém, Pará, Brazil; 2 Instituto de Ciências da Saúde, Universidade Federal do Pará, Belém, Pará, Brazil; 3 Núcleo de Medicina Tropical, Universidade Federal do Pará, Belém, Pará, Brazil; 4 Programa de Pós-Graduação em Ciências da Saúde, Universidade Federal do Amapá, Macapá, Amapá, Brazil; 5 Departamento de Psicologia Experimental, Instituto de Psicologia, Universidade de São Paulo, São Paulo, São Paulo, Brazil; 6 Universidade Ceuma, São Luís, Maranhão, Brazil; University of Melbourne, AUSTRALIA

## Abstract

We have used the Farnsworth-Munsell 100-hue (FM 100) test and Mollon-Reffin (MR) test to evaluate the colour vision of 93 subjects, 30.4 ± 9.7 years old, who had red-green congenital colour vision deficiencies. All subjects lived in Belém (State of Pará, Brazil) and were selected by the State of Pará Traffic Department. Selection criteria comprised the absence of visual dysfunctions other than Daltonism and no history of systemic diseases that could impair the visual system performance. Results from colour vision deficient were compared with those from 127 normal trichromats, 29.3 ± 10.3 years old. For the MR test, measurements were taken around five points of the CIE 1976 colour space, along 20 directions irradiating from each point, in order to determine with high-resolution the corresponding colour discrimination ellipses (MacAdam ellipses). Three parameters were used to compare results obtained from different subjects: diameter of circle with same ellipse area, ratio between ellipse’s long and short axes, and ellipse long axis angle. For the FM 100 test, the parameters were: logarithm of the total number of mistakes and positions of mistakes in the FM diagram. Data were also simultaneously analysed in two or three dimensions as well as by using multidimensional cluster analysis. For the MR test, Mollon-Reffin Ellipse #3 (u’ = 0.225, v’ = 0.415) discriminated more efficiently than the other four ellipses between protans and deutans once it provided larger angular difference in the colour space between protan and deutan confusion lines. The MR test was more sensitive than the FM 100 test. It separated individuals by dysfunctional groups with greater precision, provided a more sophisticated quantitative analysis, and its use is appropriate for a more refined evaluation of different phenotypes of red-green colour vision deficiencies.

## Introduction

Colour vision assessment is an important tool to colour vision screening or to select candidates for some specific jobs where normal trichromatic vision is desirable or mandatory (e.g., colour matching and assessment in the textiles or paint industries, grading of gemstones) [1,2]. In addition, it has been demonstrated that colour vision assessment has high diagnostic and predictive value in a variety of congenital, degenerative, and acquired visual dysfunctions [1,2]. A variety of colour vision tests was developed in more than a century of studies based in different principles and procedures. Some of them are largely used in current days by optometrist or ophthalmologists for the selection of job candidates and studies of visual dysfunctions.

Some tests use pseudoisochromatic stimuli to assure that colour discrimination is based only in chromatic differences. Pseudoisochromatic stimuli are based on the Principle of Stilling and are composed by a target and a field formed by elements varying in size and luminance and differing from each other by their colour [3]. Due to the presence of spatial and luminance noise, the colour difference is supposed to be the only cue that subjects use to discriminate the target from the field [4]. Some tests using pseudoisochromatic stimuli are widely used such as the Ishihara test [5,6], the Hardy-Rand-Rittler (HRR) test [7–9], and the Mollon-Reffin test whose commercial version is called Cambridge Colour Test [4,10–13].

The Mollon-Reffin test combines the Principle of Stilling with the Principle of Chibret of dynamically and adaptively varying the chromatic difference of target and field along different directions in colour space [14]. The Cartesian distance between target and field chromaticities in the CIE 1976 colour space represents colour contrast and the correct target discrimination decreases as the distance between target chromaticity and field chromaticity decreases until a threshold criterion is reached [4,10]. Colour discrimination thresholds can be estimated along different chromatic axes irradiating from different field chromaticities providing the means to plot the MacAdam colour discrimination ellipses for different regions of the colour space [15]. It should be noted that in the Mollon-Reffin test, differently from other pseudoisochromatic tests such as the Ishihara test, colour discrimination requires simultaneous handling of two parameters, hue and saturation, at the same luminance level. The Mollon-Reffin test was used by many research groups to study several visual dysfunctions such as daltonism [4], Parkinson disease [16,17], diabetes [18,19], mercury intoxication [20,21], Duchenne muscular dystrophy [22], multiple sclerosis [23], and alcoholism [24], among others. An adaptation of the Mollon-Reffin test was also used to investigate the perceptual interaction of colour and luminance in the identification of objects [25].

Colour arrangement tests usually consist of a certain number of patches of different hues and having the same saturation and luminance which should be arranged in the correct order starting from a reference hue [1,2]. Colour deficient subjects arrange the discs in an order that is very different from normal trichromats both in the number of mistakes that they do and the orientation of mistakes in the colour space. The most widespread used colour arrangement tests are the Farnsworth-Munsell 100-hue (FM 100) [26,27], the Farnsworth-Munsell Dichotomous D-15 or Panel D-15 (D-15) [28,29], and Lanthony Desaturated D-15 (D-15d) [30,31]. The FM 100 test is considered to be very efficient in assessing colour vision but it takes too long to perform for it to be used in some routine conditions [2]. Among the alternatives proposed by Farnsworth and others, the D-15 test which takes fourth or five times less to be applied has been the more popular choice. In addition, the D-15d test imposes a more difficult task to the subject because colour differences are smaller and can be used to a more close study of subjects that passed in the D-15 test [2].

In this work, we studied the colour vision of congenital colour deficient subjects and normal trichromats with the FM 100 and MR tests. The results obtained were first subjectively analyzed to check the feasibility of reaching a reliable diagnosis by quickly inspecting them in a screening service. The results obtained with the two tests were compared to look after possible inconsistencies. In addition, a statistical analysis was performed on the main parameters provided by both tests to check how efficiently they were able to separate congenital colour deficients from normal trichromats as well as protans from deutans. Finally, we compared the results obtained with the five colour discrimination ellipses provided by the MR test, to see which of them more efficiently separated protans from deutans.

## Methods

### Ethics Statement

This work was approved by the Ethics Committee for Research with Human Subjects of the Tropical Medicine Nucleus, Federal University of Pará, Belém, Pará, Brazil; protocol #038/2004 –CEP / NMT, date of approval 30^th^ April 2004. All subjects signed an informed consent prior to the experiments, which followed The Code of Ethics of the World Medical Association (Declaration of Helsinki) for experiments involving humans.

### Subjects

We evaluated the colour vision of 93 subjects, 30.4 ± 9.7 years old (range between 18–59 years old), 90 men and 3 women, that were previously diagnosed of red-green congenital colour vision deficiencies but had no history of additional congenital, degenerative, traumatic, toxic, or infectious diseases that could impair their visual system performance. The initial diagnostic was performed by an ophthalmologist in the State of Pará Traffic Department and was based in anamnesis, routine ophthalmological exam, and colour vision assessment of each eye. After this initial diagnosis, subjects were sent to the Federal University of Pará for further testing.

In the University laboratories, subjects’ visual acuity was monocularly measured with Snellen optotypes, aiming to evaluate the ability of fine detail discrimination at high contrasts. Only patients with minimum visual acuity of 20/25 were included in this study. Then, the subject’s colour vision was again assessed with the Ishihara Pseudoisochromatic test [32] applied separately to each eye. Finally, subjects that passed in all the inclusion criteria were then evaluated with the main colour vision tests used in this work as described below.

We have also tested 127 control subjects, 29.3 ± 10.3 years old (range between 17–60 years old), 79 men and 48 women. The subjects of the control group were chosen by applying the same inclusion criteria used for the congenital colour vision deficient and, in addition, had to have been considered normal trichromats in the initial evaluation performed with the Ishihara test. In the subsequent tests all subjects, controls or colour vision deficient, were monocularly tested using the dominant eye.

### Software

We evaluated subject’s colour vision with computerized versions of the Farnsworth-Munsell 100-Hue (FM 100) test [26] and the Mollon-Reffin (MR) test [10] developed in the Laboratory of Neurophysiology, Federal University of Pará [20,21]. The FM 100 test is a hue arrangement task using colours with the same saturation and luminance [26,27]. The MR test, commercially available as Cambridge Colour Test or CCT (Cambridge Research Systems, Rochester, England, U.K.), measures colour discrimination thresholds with stimuli having the same mean luminance and variable saturation and hue [4,10]. The system used in this work had a resolution of only 8 bits per gun, whereas the commercial version of the MR test uses 14 bits. The 8-bit version is relatively satisfactory [11], although it remains unknown whether a 14-bit system would offer better diagnosis.

Software was written using C++ programming language, OFS/Motif 1.1, AIX-Windows R4, and IBM-GL graphic library, all for the AIX 3.2.x computer environment [20,21]. It was implemented in an IBM POWERStation RISC 6000 320H (IBM Corporation, New York, NY, U.S.A.). The visual stimuli were generated using IBM GT4-3D graphic adapters, 24 bits / 8 bits per gun, and were displayed in an IBM 6091 19i CRT colour monitor, 1280 x 1024 pixels, 81.32 kHz horizontal refresh rate, 77-Hz vertical frame rate. Luminance and chromaticity were measured with a Tektronix J16 digital photometer equipped with a 1° luminance probe Tektronix J6523-2 (Tektronix, Beaverton, OR, U.S.A.) and a CS-100A chromameter (Konica Minolta, Mahwah, NJ, U.S.A.). The CIE 1976 coordinates of monitor guns were: red, u’ = 0.427, v’ = 0.523; green, u’ = 0.115, v’ = 0.559; blue, u’ = 0.165, v’ = 0.163.

### FM 100 Test

#### Subjects

The FM 100 test was applied to 91 colour vision deficient subjects, 30.5 ± 9.8 years old (range: 18–59 years old), 88 men and 3 women. The remaining 2 colour vision deficient subjects from our sample had limited time available for this study and did not perform this test. The results were compared with those from 91 age-matched control subjects from our sample. All subjects were monocularly tested after an initial 15 min adaptation to the room 0.02 cd / mm^2^ background luminance.

#### Stimulus

The FM 100 test comprised one set of 22 colour patches and three sets of 21 colour patches, equally spaced on the CIE 1976 chromaticity diagram, all having the same luminance (42 cd/m^2^) and saturation (30% purity), differing in hue, which should be arranged by the subject in a progressive hue order. The colour patches measured 1 deg^2^ and were presented in the CRT screen placed at 1 m of distance from subject’s eye.

#### Procedures

Four sets of colour patches were sequentially presented to the subject. The colour patches were arranged in the four sets according to their hue: magenta/orange hues, yellow/green hues, blue/purple hues, and purple/magenta hues. At the beginning of each set, the complete correct sequence was initially presented to the subject and after 1 min period of observation, the patches were randomly distributed in the screen. The colours at the ends of each set were kept in their position, to serve as anchors. The subject’s task was to rearrange the patches following the initial order that was previously shown to him. There was no limit of time imposed to the subject. The subject repeated the task four times and the results were averaged to provide the final score for the subject’s colour vision performance.

#### Analysis

Two quantitative parameters were relevant to evaluate subject’s performance in this test. First, a numerical score based on the number of errors made by the subject in the ordering task was provided [1,27]. The subject’s score for each hue axis, *Score*_*i*_ for *i* = {1 a 85}, was estimated as the sum of the absolute values of the differences between the number of the hue placed by the subject in the position *i* (*n*_*i*_) and the numbers of the hues placed in their left and right neighbour positions (*n*_*i-1*_ and *n*_*i+1*_, respectively):

$$Score_{i}\;=\left|n_{i}-n_{i-1}\left|+\right|n_{i}-n_{1+i}\right|$$ (1)

Using Eq 1, a *Score*_*i*_ = 2 means that the subject placed the *hue*_*i*_ in the correct position. The *Total Error* was estimated as the sum of all individual scores for the 85 axes minus 170. Thus, for a subject that placed all the hues in their right positions, the *Total Error* was equal to zero.

$$Total\;Error=(\sum_{i=1}^{85}Score_{i})-170$$ (2)

For statistical comparisons, we have used the logarithm of the *Total Error*.

Second, as errors may concentrate in one of the colour confusion axes, protan and deutan for the congenital colour vision deficient subjects that were studied in this work, we quantified the type of error by measuring the right and left central points of the error distribution in the Farnsworth-Munsell diagram [1,27]. The left central point corresponded to a hue axis between *n*_*1*_ and *n*_*43*_ and the right central point corresponded to a hue axis between *n*_*44*_ and *n*_*85*_ in the Farnsworth-Munsell diagram, in such a way that the cumulative score in the clockwise direction was equal to the error median in the respective hemisphere.

### MR Test

#### Subjects

The MR test was applied to about half of the colour vision deficient subjects that were studied, 47 subjects, 31.8 ± 10.1 years old (range between 18–59 years old), all men. The remaining 46 subjects were not available for this part of the study. The results were compared with those from 47 age-matched control subjects from our sample. Subjects were monocularly tested after an initial 15 min adaptation to the room 0.02 cd / mm^2^ background luminance.

#### Stimulus

Stimuli consisted of a target presented in a field of different chromaticity but similar spatial and luminance structure [4]. The target had the shape of a Landoldt C measuring 4.3° of external diameter, 2.2° of internal diameter, and having a 1° gap. Colour discrimination thresholds were determined based on the subject’s ability to discriminate the target orientation, that is, if the gap was pointing to the upper, lower, right, or left sides of the screen. The discrimination was performed in the presence of spatial and luminance noise to prevent the subject to use other cues than chromaticity difference to discriminate the target from the field. For this purpose, stimulus target and field consisted of spatially discrete circular patches of varying size (ten different sizes from 0.2–0.6 degrees of diameter) and luminance (six different luminance levels from 12–20 cd/m^2^), randomly presented on a black background.

#### Procedures

The subjects were placed at 3 m from the screen and were instructed according to Mollon and Regan [33]. The stimulus was presented for 1.5 s. The circular patches that formed the field had the same chromaticity which remained constant across the trials. The circular patches that formed the target also had the same chromaticity but it changed from trial to trial, approaching the field chromaticity through a binary staircase until the discrimination threshold was reached [4]. The chromaticity difference between target and field along a given direction of the colour space was adaptively increased or decreased according to the subject's performance. Testing was terminated after eleven reversals and the threshold was taken as the mean chromaticity value of the last six reversals. Alternatively, testing was terminated after five failures to detect the most saturated stimulus and the threshold was then considered as an arbitrary maximum value. Five discrimination ellipses were obtained in a single session but the subjects could rest during 5 min intervals on demand along the session.

#### Analysis

Using this approach, twenty chromaticity discrimination thresholds were estimated around five points located in the CIE 1976 colour space. Data points representing measured thresholds were fitted by ellipses using a routine written in Visual Basic for Application of Excell 2003 (Microsoft Corporation, Redmond, WA, U.S.A.). In this way, five Mollon-Reffin’s chromaticity discrimination ellipses were generated [4]. These ellipses are MacAdam ellipses generated by the MR test. The ellipses’ centres corresponded to the field chromaticities and were, in (u’, v’) coordinates: Ellipse #1, (0.215, 0.531); Ellipse #2, (0.219, 0.481); Ellipse #3, (0.225, 0.415); Ellipse #4, (0.175, 0.485); Ellipse #5, (0.278, 0.472).

### Additional Tests

A subsample of subjects (n = 20) also performed the red-green Rayleigh match in a HMC anomaloscope (Oculus Optikgeräte GmbH, Wetzlar, Germany) and had blood samples taken for genetic molecular analysis. Genetic screening was performed using real time quantitative polymerase chain reaction (RT-PCR) and used to estimate the relative number of L and M cone opsin genes. It will be fully reported in a different publication (D. M. O. Bonci and colleagues, personal communication). The results of these additional analyses were used to check the reliability of the FM 100 and MR tests.

### Determination of Subject’s Phenotype

#### Subjective analysis

This procedure was performed to verify how precise ophthalmologists or optometrists could quickly analyze the results from FM 100 test and MR test in their daily routine. Six observers performed a subjective analysis of the results by inspecting their graphical representations for the FM 100 and MR tests. Each observer was instructed about what to expect from subject’s graphical results. In addition, they were allowed to previously inspect examples obtained from normal trichromats, protans, and deutans. For the FM 100 test, the observers were told how the results were plotted in the polar coordinates of the Farnsworth-Munsell diagram and that they should pay attention to the magnitude and orientation of the largest scores, as well as the numerical value of subject’s *Total Error*. For the MR test, the observers were told how the results were plotted in Cartesian coordinates of the CIE 1976 colour space and that they should pay attention to the sizes and orientations of the Mollon-Reffin ellipses, and that they should use the information provided by all five ellipses that were measured. Then, the observer was asked to classify each subject in one of the three categories—normal trichromats, protans, and deutans—inspecting the results of the FM 100 test and MR test one at each time. During the process, the observers had no indication which subject they were classifying and they did the two sets of classifications in a randomly manner. In this analysis, the final classification of each subject was that provided by the majority of observers and it was separately considered for each test.

#### Statistical analysis

The results of the FM 100 test were plotted in polar coordinates using the Farnsworth-Munsell diagram while the Mollon-Reffin ellipses were plotted in Cartesian coordinates using the CIE 1976 colour space. In addition, we also displayed the results in three dimensional Cartesian plots for simultaneous analysis of different parameters using the software Matlab v. 7.11.0.584 (R2010b) (MathWorks, Natick, MA, U.S.A.).

The parameters measured in the FM 100 test and MR test were submitted to statistical analysis using BioEstat 3.0 software [34]. Kruskal-Wallis one-way analysis of variance by ranks and Student-Newman-Keuls method as post hoc test were used for comparisons between protans, deutans, and controls. Cluster analysis was performed employing Ward’s method and Euclidian distances employing Statistica for Windows 5.0 software (StatSoft, Tulsa, OK, U.S.A.).

The statistical norms for the performance of congenital colour vision deficient subjects in the MR test were estimated. As the number of protans and deutans that did the MR test in this study was limited, the resulting statistic norms should be accepted as preliminary. In this analysis, the tolerance interval was estimated. It represents the range of values where a *p* percentage of the studied population is contained. In this study, *p* was stipulated as corresponding to 90% of the population. As the tolerance interval was based in data obtained from a limited sample instead of population’s parameters, it was not possible to be 100% sure that it contained the specified *p* proportion of the population. Thus, to estimate the tolerance interval, the degree of confidence γ was also used, considering 95% for its desired value [35]. Using *p* = 0.90 and γ = 0.95, the extreme values for the tolerance interval could then be estimated [21,36]. In this work, the two-tailed upper tolerance limit (*uTL*) and lower tolerance limit (*lTL*) were estimated using the following equations:

$$uTL=\bar{X}+ks$$ (3)

$$lTL=\bar{X}-ks$$ (4)

$$k=\sqrt{\frac{\left(N-1\right)\left(1+\frac{1}{N}\right)z_{\left(1-p\right)/2}^{2}}{\chi_{\gamma ,N-1}^{2}}}$$ (5)

Where N,$\bar{X}$, and *s* were the sample’s size, mean, and standard deviation, respectively; *k* was a parameter estimated in such a way that the tolerance interval comprised a *p* proportion of the population with γ degree of confidence;$\chi_{\gamma ,N-1}^{2}$ was the critical value of the χ^2^ distribution with *N*-1 degree of freedom and probability γ; and *Z*_(1−*p*)/2_ was the critical value of the normal distribution with probability (1-*p*)/2.

## Results

### Subjective Analysis

All subjects tested were classified by six observers by inspecting the graphic results of their performance in the FM 100 test and the MR test, results of each test at time. All controls were classified as normal trichromats by all six observers in both tests. All congenital colour vision deficient subjects were classified as having red-green colour vision deficiencies by all six observers either using the FM 100 test results or the MR test results.

For the large majority of colour vision deficient subjects, four to six observers agreed in their classification as protans or deutans. For the FM 100 test, the majority of observers agreed about the classification of 81 subjects (six about 42 subjects, five about 22 subjects, and four observers agreed about 17 subjects). For the remaining 10 subjects there was agreement only among half of observers and they were allocated in the protan or deutan groups after a discussion of their results by the group of observers.

For the MR test, using together the results from all five colour discrimination ellipses, the six observers agreed about the classification of all 47 colour vision deficient subjects as protans or deutans.

There were five disagreements between results obtained with the two tests: two subjects classified in the MR test as protans were classified as deutans in the FM 100 test (subjects P015 and P062), and three subjects classified in the MR test as deutans, were classified as protans in the FM 100 test (subjects D001, D007, and D092). The inspection of the results of these five subjects revealed that they indeed were difficult to classify by the FM 100 test. On the other hand, the MR results were clearer. It was possible to further investigate the colour vision of two of these subjects using anomaloscopy and molecular genetic analysis and the results provided by the MR test were confirmed by both techniques (subjects P015 and D092).

Anomaloscopy and genetic analysis provided similar classification for all 20 congenital colour vision deficient subjects tested with these techniques. They confirmed the results obtained with the psychophysical tests for 17 subjects. They diverged from the FM 100 test in three subjects (subjects P015, D044, and D092) and from the MR test in a single subject (subject D044). As mentioned above, P015 was classified as deutan by the FM 100 test and protan by the MR test, anomaloscopy, and genetic analysis, while D092 was classified as protan by the FM 100 test and deutan by the MR test, anomaloscopy, and genetic analysis. D044 was classified as deutan by both the FM 100 test and the MR test, but anomaloscopy and genetic analysis indicated that he was protan. The inspection of the results of D044 revealed that he was very difficult to classify him either as protan or deutan with the FM 100 test or the MR test. In the MR test, Ellipses #1, #2, and #5 had a deutan orientation while ellipse #3 and #4 had a clear protan orientation in the CIE 1976 colour space.

Further analysis of the results used the classification provided for the test considered, but the final classification adopted for controls, protans, and deutans was that provided by the MR test, when available, or that provided by the FM 100 test when the former was unavailable.

### Statistical Analysis—FM 100 Test

**Fig 1:**
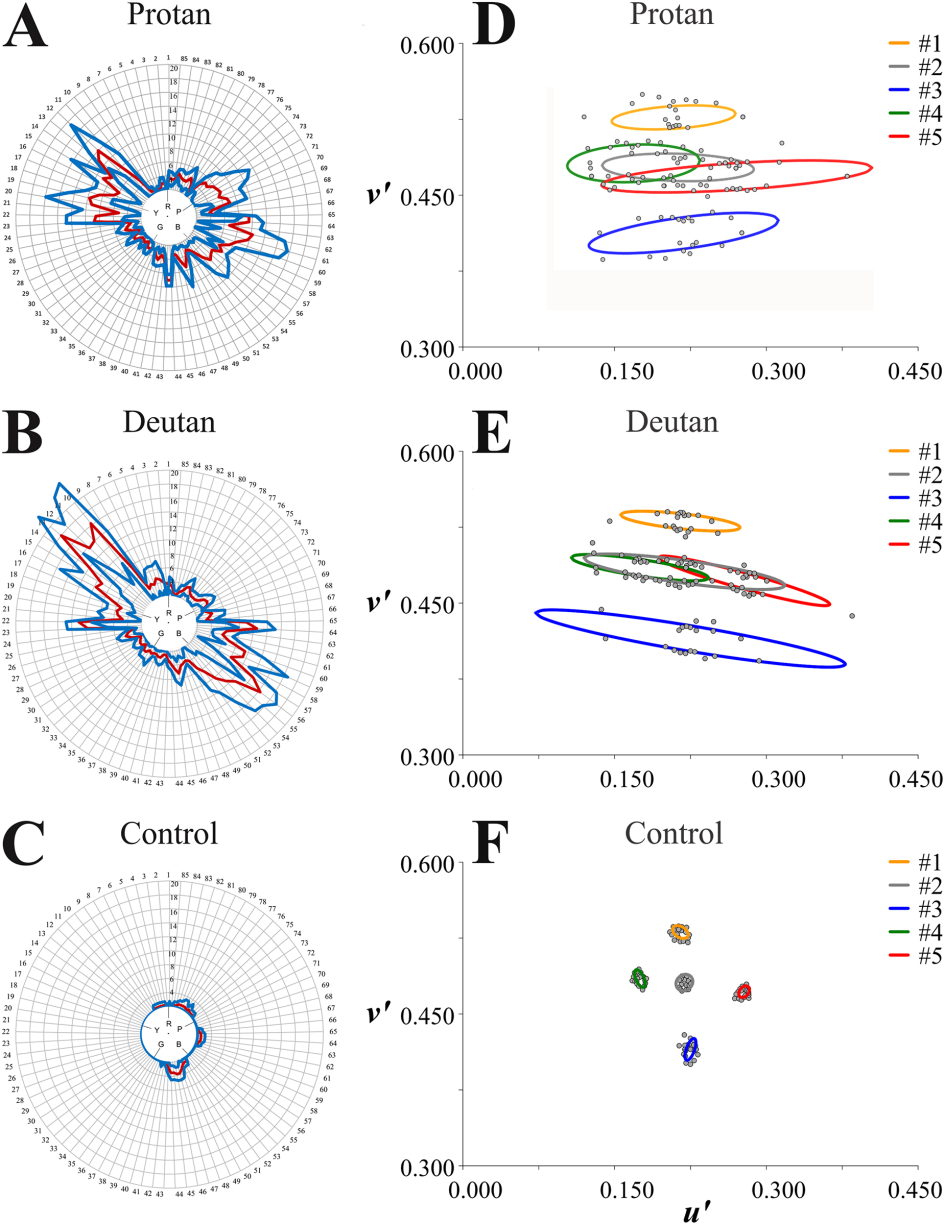
FM 100 results compared with MR results. The three phenotypes studied in this work are illustrated with results obtained from a single subject from each group. (**A,D**) Protan subject P005. (**B,E**) Deutan subject D073. (**C,F**) Normal trichromat subject C178. (**A-C**) FM 100 results. The red and blue contours represent means and standard deviations, respectively, for the *Score*_*i*_ (n = 4 trials). The colour vision deficient subjects made more mistakes than the control subject. In addition, the mistakes made by the protan and deutan subjects had specific orientations in the FM diagram. (**D-F**) MR results were plotted in the *u’v’* coordinates of the CIE 1976 colour space. Data points were fitted with ellipses to generate the Mollon-Reffin colour discrimination ellipses. Both the protan and deutan had larger colour discrimination ellipses than the normal trichromat. In addition, protan and deutan thresholds were elongated along their respective colour confusion lines, making their ellipses more elongated than the ellipses of the normal trichromat.

**Fig 2:**
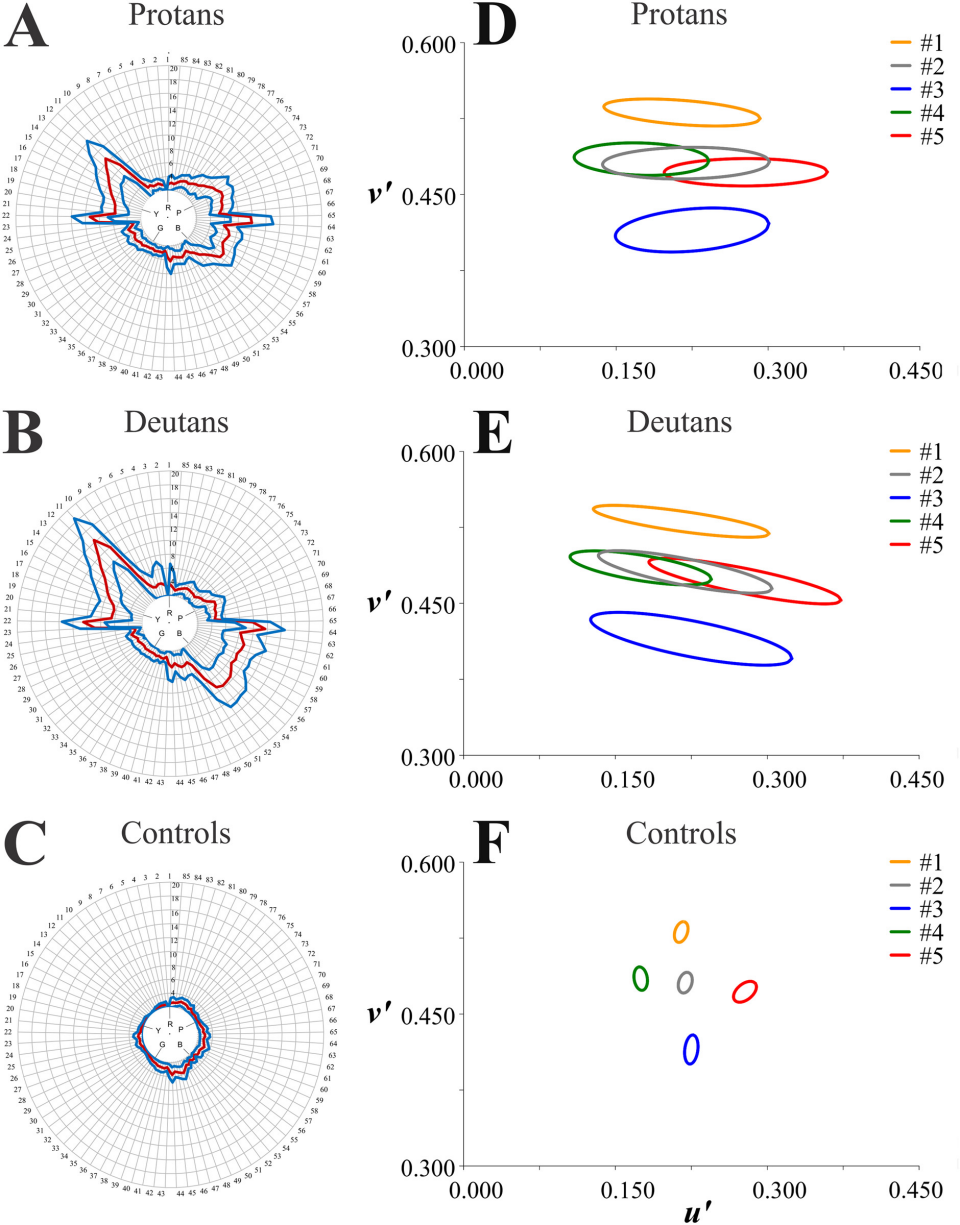
FM 100 results compared with MR results. The phenotypes studied in this work are illustrated with average results for the three groups. Subjects were classified as (**A,D**) protans, (**B,E**) deutans, and (**C,F**) controls according to their performance in the FM 100 test or the MR tests.

Figs 1 and 2 show results obtained with the FM 100 test displayed in the Farnsworth-Munsell diagram. Mistakes made by subjects were plotted in polar coordinates: radial directions represent the main hues (R, red; Y, yellow; G, green; B, blue; P, purple) and five series of intermediate hues numbered from 1 to 85; distances from the centre represent the *Score*_*i*_ for each hue *i* and correspond to the mistakes made by the subjects in the hue arrangement (*Score*_*i*_ = 2 means no mistake for the *i* hue).

Fig 1A–1C shows results for representative individuals of the three groups studied with the FM 100 test: a protan (P005), a deutan (D073), and a normal trichromat (C178). Each subject repeated the test four times and the results were displayed as means (red contours) and standard deviations (blue contours) around the hue circle. Both the protan and the deutan made more mistakes than the normal trichromat, that is, the *Total Erro*r was larger for the two subjects with congenital colour vision deficiencies than for the control subject. In addition, the regions of the Farnsworth-Munsell diagram where the protan and deutan made more mistakes were distinct and could be characterized by using the values for the *Left Central Point* and *Right Central Point* (see below).

Fig 2A–2C shows average results in the FM 100 test for each group studied. As described in the previous section, according to their performance in the FM 100 test and independently of their results in the MR test, the subjects were classified as (A) protans (n = 44), (B) deutans (n = 47), and (C) normal trichromats (controls) (n = 91). In the figure, red and blue contours represent grand means and standard deviations, respectively, for mistakes made by each group of subjects—grand means estimated from means of the four trials performed by each subject. The *Total Error* was 243 ± 68 for protans, 297 ± 88 for deutans, and 53 ± 28 for controls. The log (*Total Error*) was: 2.37 ± 0.12 for protans, 2.46 ± 0.09 for deutans, and 1.64 ± 0.31 for controls. As a group, both protans and deutans made more mistake in the FM 100 test than normal trichromats.

**Fig 3:**
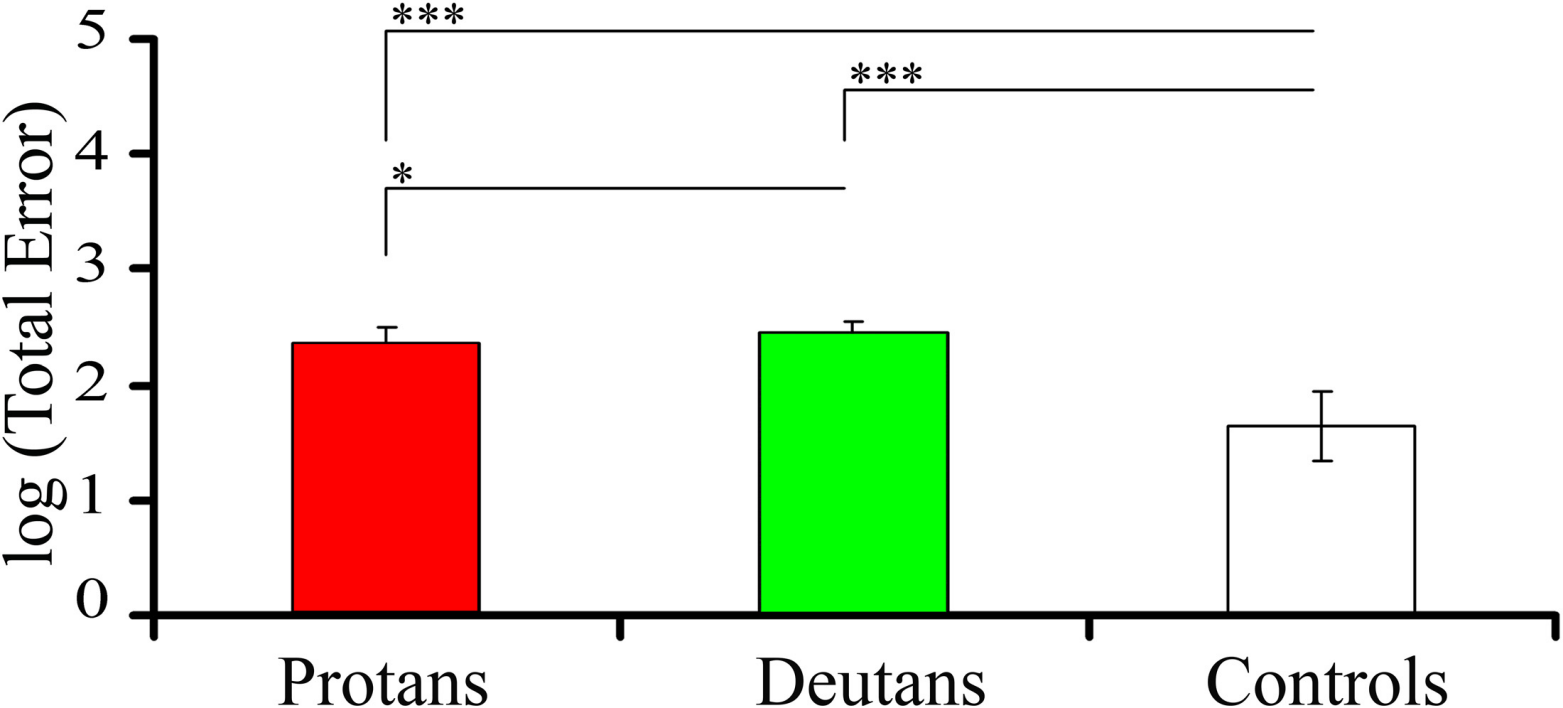
Statistical comparisons of the FM 100 results: total number of mistakes. Comparisons between the number of mistakes made by protans (n = 44), deutans (n = 47), and controls (n = 91). The number of mistakes was taken as the logarithm of the *Total Error*. The columns and error bars represent, respectively, means and standard deviations for the three groups. There were statistical significant differences between the number of mistakes made by protans or deutans versus controls, as well as between protans and deutans. Significant differences: *p < 0.05; ***p < 0.0001; Kruskal-Wallis.

Fig 3 shows the statistical comparison between the log (*Total Error*) for the three populations. There were very significant differences between protans and controls (p < 0.0001) and deutans and controls (p < 0.0001). There was also significant difference between protans and deutans mostly due to some deutans with very large log (*Total Error*) (p < 0.05).

Once colour vision deficient subjects and controls were distinguished by the number of mistakes they made in the hue arrangement, the region of the Farnsworth-Munsell diagram where they made more mistakes could be used to separate them in protans and deutans. The *Left Central Point* was 17 ± 2 for protans, 15 ± 2 for deutans, and 30 ± 6 for controls, while the *Right Central Point* was 64 ± 1 for protans, 60 ± 1 for deutans, and 59 ± 5 controls, respectively. The difference between protans and deutans for the *Left Central Point* was significant (p < 0.01) and the difference between them for the *Right Central Poin*t was very significant (p < 0.0001). In addition, protans differed from controls for both the *Left Central Point* and *Right Central Point*, and deutans differed from controls for the *Left Central Point* (p < 0.0001). However, there was no significant difference between deutans and controls for the *Right Central Point* (p > 0.5).

Thus, in the analysis of the FM 100 results, the *Total Error* was a good criterion to separate congenital colour vision deficient subjects from controls while the *Right Central Point* was the best criterion to distinguish between protans and deutans. The reason for this difference between central points is that hues *i* = 52 to 73 of the right side of the Farnsworth-Munsell diagram were located in a region of the colour space where protan and deutan confusion lines intercept each other at large angles and these two kinds of subjects see colours very differently from each other in this region [2].

**Fig 4:**
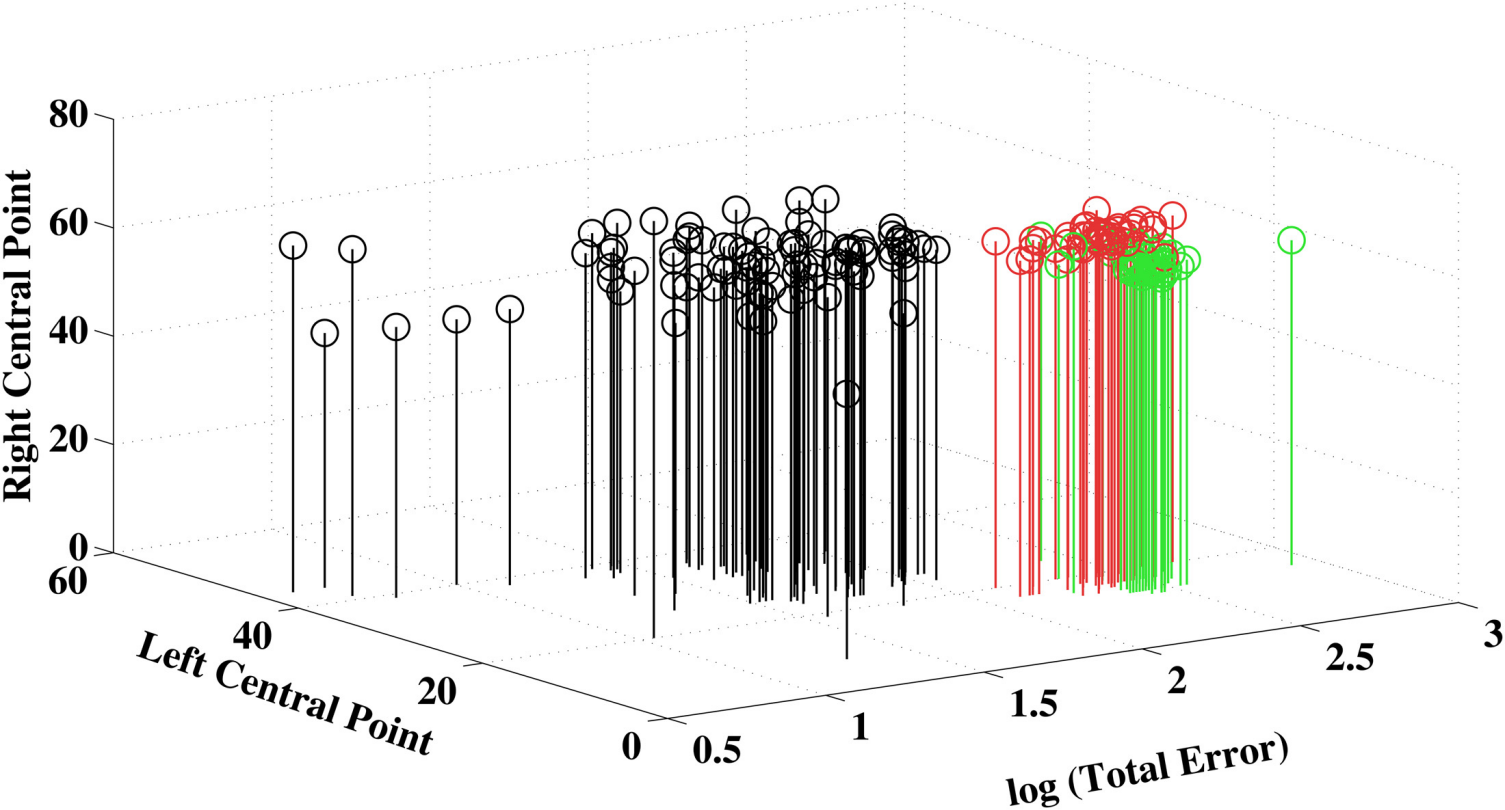
FM 100 results visualized in three dimensional Cartesian plots. The values of log (*Total Error*), *Left Central Point*, and *Right Central Point* were plotted for all subjects that performed the FM 100 test. The values for *Total Error* completely separated congenital colour vision deficient subjects from controls. Introduction of central points in this analysis separated protans from deutans mostly due to distinct higher values of *Right Central Point* and slightly lower values of *Left Central Point* for protans in relation to deutans. A few deutans intruded in the protan region of the plot (see text for further discussion of this issue).

Fig 4 shows the results for the three parameters of the FM 100 test plotted together. Protans and deutans were entirely separated from controls due mainly to the values for log (*Total Error*) and *Left Central Point*. Protans and deutans formed a single cluster in a plan having log (*Total Erro*r) and *Left Central Point* as coordinates, with some segregation between them in their projection to the *Left Central Point* axis. The introduction of the *Right Central Point* as the third coordinate separated the majority of protans from deutans due to distinct higher values of *Right Central Point* of protans. A few deutans remained intruded in the protan region of the plot. Thus, both in the subjective and statistical analyses, there was a minority of congenital colour deficient subjects whose classification as protans or deutans remained doubtful in the FM 100 test.

### Statistical Analysis—MR Test

Fig 1D–1F shows results obtained with the MR test displayed in the CIE 1976 u’v’ colour space. Colour discrimination thresholds were measured along 20 directions irradiating from references points located in 5 different regions of the colour space [4]. Data points representing measured thresholds were then fitted with ellipses to generate the Mollon-Reffin colour discrimination ellipses. Fig 1D–1F illustrates results obtained from individual subjects from the three groups: a protan (P005), a deutan (D073), and a normal trichromat (C178). Both the protan and deutan had larger colour discrimination thresholds along their respective colour confusion lines, making their ellipses larger and more elongated than the ellipses of the normal trichromat.

As already described, subjects were then classified by six observers as protans (n = 18), deutans (n = 29), and normal trichromats (controls) (n = 47) according to their graphic results in the MR test independently of their results in the FM 100 test. The colour discrimination threshold grand means for each group were then fitted with ellipses and presented in Fig 2D–2F as average colour discrimination ellipses for protans (A), deutans (B), and controls (C). Protans and deutans ellipses were much larger than those of controls and elongated in the directions of the protan and deutan confusion lines towards the protan and deutan copunctal points, respectively.

**Fig 5:**
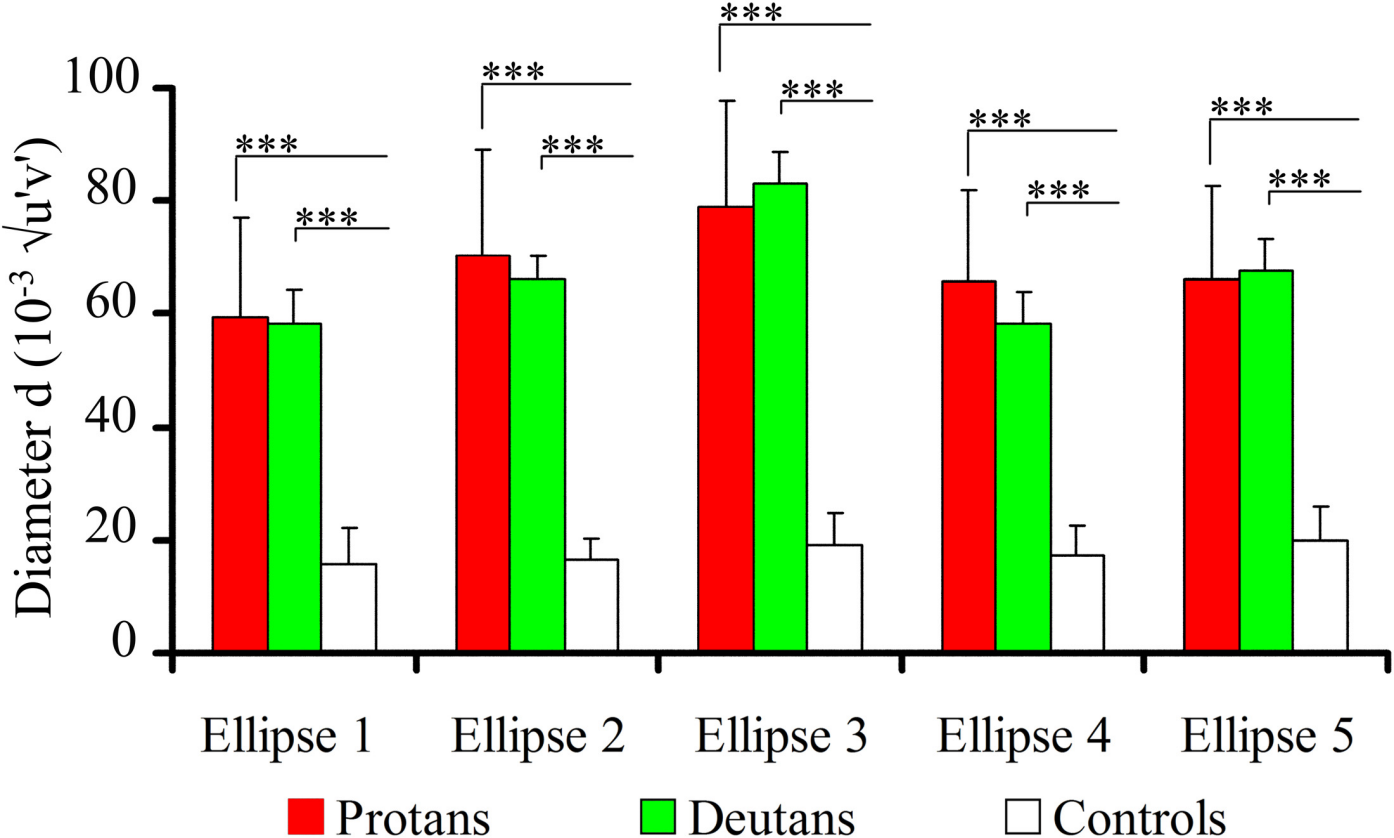
Statistical comparisons of results obtained with the MR test: sizes of the Mollon-Reffin ellipses. Comparisons between the sizes of colour discrimination ellipses for protans, deutans, and controls. Both protans and deutans had ellipses significantly larger than controls. There were no statistical differences between protans and deutans. Significant differences: ***p < 0.0001; Kruskal-Wallis.

**Fig 6:**
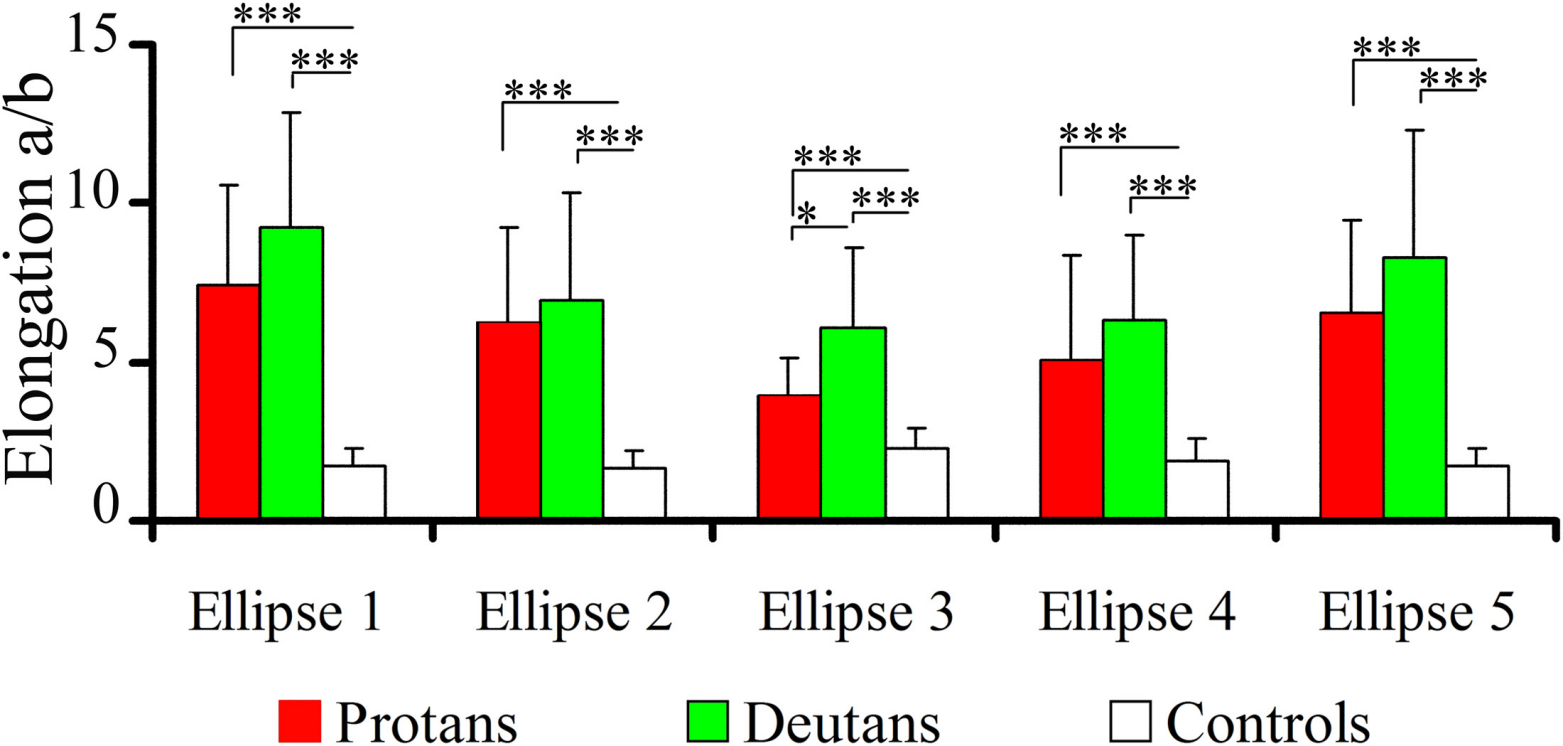
Statistical comparisons of results obtained in the MR test: elongations of the Mollon-Reffin ellipses. Comparisons between the elongations of colour discrimination ellipses for protans, deutans, and controls. Both protans and deutans had ellipses significantly more elongated than controls. There were no statistical differences between protans and deutans, except for Ellipse #3. Significant differences: *p = 0.05; ***p < 0.0001; Kruskal-Wallis.

Results obtained with the MR tests were further analyzed to perform statistical comparisons between the three subject groups for the following ellipse parameters: size, elongation, and orientation. Data for each one of the five ellipses were separately analyzed. Fig 5 shows the results for ellipse size expressed as the diameter of the equivalent circle *d*, in 10^−3^ √*u*’*v*’ unities. Both protans and deutans had all five ellipses significantly larger than controls (p < 0.0001). There were no significant statistical differences between protans and deutans for the sizes of all five ellipses (p > 0.5). Fig 6 shows the results for ellipse elongation, *a*/*b*, taken as the ration between the length of ellipse long axis (*a*) and the length of ellipse short axis (*b*), respectively. Both protans and deutans had all five ellipses significantly more elongated than controls (p < 0.0001). For all five ellipses, the differences of elongation between protans and deutans were no significant (Ellipses #1, #4, and #5, p > 0.1; Ellipse #2, p > 0.5) or had low significance level (Ellipse #3, p = 0.05).

**Fig 7:**
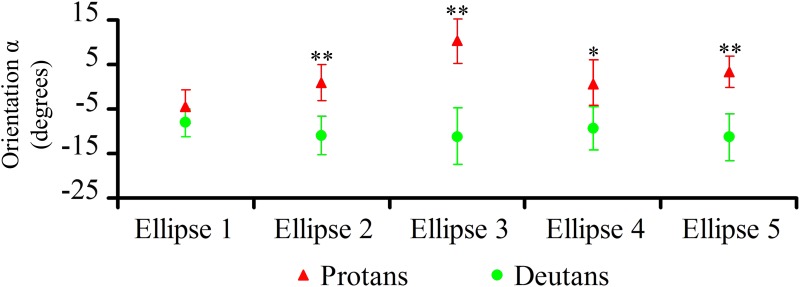
Statistical comparisons of results obtained with the MR test: orientations of the Mollon-Reffin ellipses. Comparisons between the orientations of colour discrimination ellipses for protans, deutans, and controls. There were significant statistical differences between protans and deutans for *α* of Ellipses 2–5. The largest difference for *α* between protans and deutans occurred in the Mollon-Reffin Ellipse #3 (see text for the explanation). Significant differences: *p < 0.05; **p < 0.01; Kruskal-Wallis. Differences between protans and controls as well as deutans and controls were not illustrated.

Once ellipse size and elongation were used to segregate congenital colour deficient subjects from controls, ellipse orientation could be used to discriminate between protans and deutans. Fig 7 shows the results for ellipse orientation α, in degrees, taken as the angle formed by the right half of ellipse long axis with the u’ axis of the CIE 1976 colour space. For clarity, only data for protans and deutans are illustrated. Protans and deutans significantly differed from each other in ellipse orientation for four out of five ellipses: Ellipse #4, p < 0.05; Ellipses #2, #3, and #5, p < 0.01). The largest difference was for Ellipse #3 where α was +10.3 ± 4.9 degrees for protans and -11.1 ± 6.3 for deutans. The angular differences between protans and deutans for Ellipses #1 to #5 were 3.6°, 10°, 21.4°, 8.5°, and 8°, respectively. Table 1 shows the population statistics for the Mollon-Reffin ellipses orientation. Due to the relatively small number of protans and deutans tested in this work, these norms should be regarded as provisional.

**Table 1:** Population statistics for the MR test: angle α between the MR ellipse long axis and the *u’* axis of the CIE 1976 colour space.

			Angle of the ellipse long axis (degrees)				
Group	n	Parameter	Ellipse #1	Ellipse #2	Ellipse #3	Ellipse #4	Ellipse #5
Protan	18	UTL	4.42	10.31	21.95	13.16	11.75
		Mean	-4.40	0.93	10.27	0.70	3.44
		LTL	-13.22	-8.46	-1.40	-11.76	-4.86
Deutan	29	UTL	-1.46	-1.48	2.45	1.76	-0.22
		Mean	-8.04	-10.94	-11.08	-9.21	-11.35
		LTL	-14.62	-20.39	-24.62	-20.18	-22.48

UTL, upper tolerance limit. LTL, lower tolerance limit.

In addition (not illustrated), there were consistent significant differences in the ellipse orientation between protans and controls as well as between deutans and controls for Ellipses #1, #2, #4, and #5 at different significance levels, varying from p < 0.001 to p < 0.0001. The exception was the difference between protans and controls for Ellipse #3 that had not reached the significance level (p > 0.5).

**Fig 8:**
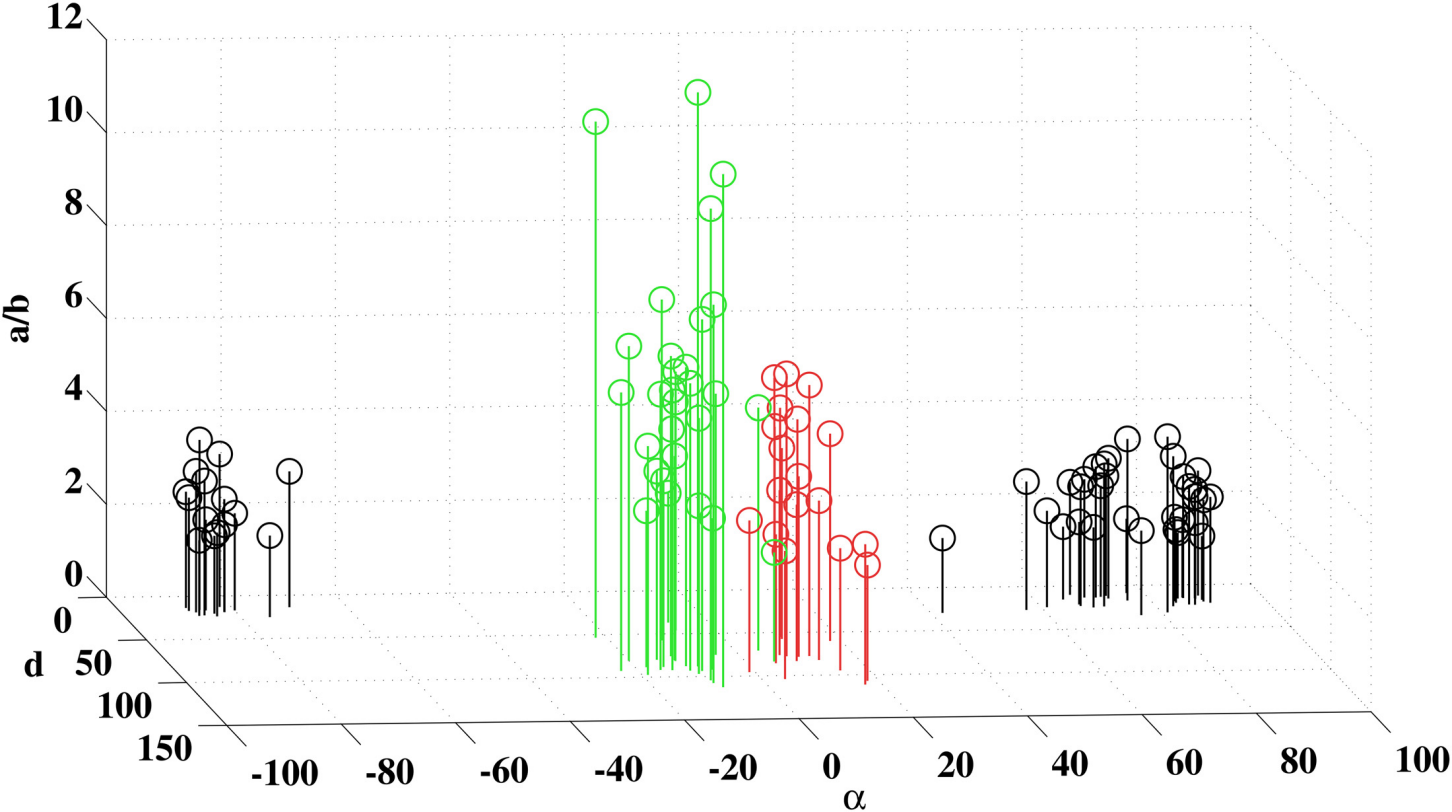
Results of the MR test visualized in three dimensional Cartesian plots. For this plot, only the results for the Mollon-Reffin Ellipse #3 were used. The values of angle *α* (orientation in degrees), diameter *d* (diameter of the circle with equal area in 10^−3^ √*u’v*’ unities, and ratio *a/b* (inclination) were plotted for all subjects that performed the MR test: protans (red bars), deutans (green bars), and controls (black bars). Ellipse #3 data completely separated colour vision deficient subjects from controls: protans and deutans had larger, more elongated, and less inclinated Ellipses #3 than controls. In addition, Ellipse #3 data separated protans for deutans almost completely, mostly due to the large difference in the ellipse orientation: protans had *α* between -1.1° and +18°, while the majority of deutans had *α* between -4.5° and -22.9°. Two subjects classified as deutans using data from all MR ellipses were plotted in the protan region of the figure (see text for more details).

The orientation (*α*), size (*d*), and elongation (*a*/*b*) were analyzed in three-dimensional plots for each ellipse. For short, only Ellipse #3 plot is shown in Fig 8 once the results for this ellipse not only were efficient to discriminate between congenital colour vision deficient subjects and control subjects but also were more efficient to discriminate between protans and deutans than the results for the other four ellipses. As expected, Fig 8 shows two distinct clusters. First, a cluster located in the central values of ellipse orientation (-25° < α < 20°) comprised all protans and deutans and none of controls. Second, a cluster located in the extremes values of ellipse orientation (-90° < α < -70° ∩ 90° > α > 40°) comprised all controls and none of the congenital colour deficient subjects. The central cluster also differs from the lateral cluster for its larger values of *d* and generally larger values of *a*/*b*.

In addition, the central cluster could be divided in two distinct regions. First, a region located on the left side of ellipse orientation values (-25° < α < -4°) comprised almost all deutans and none of protans. Second, a region located on the right side of ellipse orientation values (-2° < α < 20°) comprised all protans and only two deutans (D006 and D044). The distinction was provided essentially by difference in the values of *α*. The values of *d* and *a*/*b* for protans and deutans largely overlap.

Two deutans, D006 and D044, had Ellipse #3 angles in the protan zone: 5° and 4°, respectively (see text for more details about these two subjects). D044 was classified as deutan by the FM 100 test and the MR test (Ellipses #1, #2, and #5), but was classified as protan by the MR test (Ellipses #3 and #4), anomaloscopy, and genetic analysis. Thus, this subject was probably protan and its position in the protan region of Fig 8 is correct. D006 was classified as deutan by the FM 100 test and MR test (Ellipes #1, #2, #4, and #5), but could have been classified as protan by the MR test if only the Ellipse #3 data were used as it was done in Fig 8. Unfortunately, Ellipse #3 data were very noise and the position of this subject in the protan region of Fig 8 was probably an artifact.

**Fig 9:**
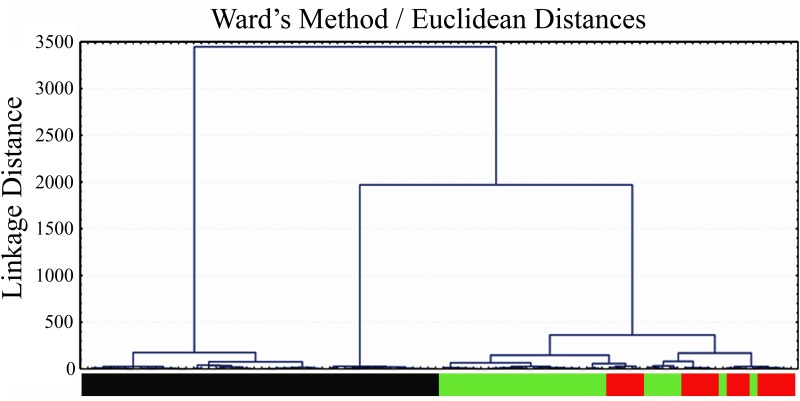
Dendrogram for MR test results. For this plot, only the results for the Mollon-Reffin Ellipse #3 were used. The dendrogram was generated using the Method of Ward and *d*, *a/b*, and *α* as parameters. The linkage distances were expressed as Euclidean distances. Ellipse #3 data split the subjects in 3 major groups and 11 subgroups which completely separated colour vision deficient subjects from controls: subgroups 1–4 comprised all controls; subgroups 5–7 and 9 comprised only deutans; subgroups 8, 10, and 11 comprised all protans plus two deutans, D006 and D044 (see text for more details about these two subjects).

A cluster analysis was also performed using data from all ellipses or each ellipse separately. The best segregation of subjects in clusters was obtained when using data from Mollon-Reffin Ellipse #3 (Fig 9). The following parameters were used: diameter *d*, elongation *a/b*, and orientation *α* (as defined in Figs 5 and 7). The dendrogram was generated using the Method of Ward and *d*, *a/b*, and *α* as parameters. The linkage distances were expressed as Euclidean distances. Subjects were classified according to their results in the MR test only: protans (red bars, n = 18), deutans (green bars, n = 29), and normal trichromats (black bar, n = 47). Ellipse #3 data split subjects in 3 major groups and 11 subgroups which completely separated colour vision deficient subjects from controls: subgroups 1–4 comprised all controls; subgroups 5–7 and 9 comprised only deutans; subgroups 8, 10, and 11 comprised all protans plus two deutans, D006 and D044. These were the same two subjects that intruded in the protan region of Fig 8 three-dimensional plot (see above for more details about these two subjects).

Comparing the several parameters provided by both tests and using r ≥ 0.7 correlation criteria, there were correlation only for Ellipse 3 a/b provided by the MR test and the location of the right central point provided by the FM100 (r = 0.91688, p = 0.00007). Thus, correlation between the results of both tests was rather low, probably owing to the different nature of the tasks.

## Discussion

In this work we assessed subjects’ colour vision with two largely used colour vision test: a computer-based version of the FM 100 colour arrangement test which is based in the discrimination of patches bearing different hues and similar saturation and luminance, and the MR pseudoisochromatic adaptive test which is based in the discrimination of targets from fields with different hues and saturations and similar luminance. Both the FM100 test and MR test have been largely used as quantitative tests as well as to provide a type diagnosis for congenital colour deficient subjects. The FM 100 test that was developed by Farnsworth [26] and largely used since then was implemented in a collection of printed colours mounted in “physical” supports while in this work we used a computer-based version of the test. We have not directly compared our computer-based version with the original FM 100 test, but data comparing computer-based and original versions of the FM 100 are available demonstrating the similarity of information provided by both versions [37]. Ghose and colleagues showed a good correlation between the two versions of the FM 100 for Total Error and colour deficiency classification and concluded that the computer-based FM 100 test was an effective and rapid method for detecting, classifying, and grading colour vision anomalies [37]. The version of the MR test used in this work was based in the published material by Regan, Reffin, and Mollon [4] and used a very similar configuration [20]. Statistical norms for this test was published using data from our version and from the commercial CCT version [11]. The results of this work for normal trichromats, protans, and deutans are very similar to those previously published by Mollon and colleagues [4,10,38].

In this work, observers were asked to classify the tested subjects through a quick inspection of the FM 100 results and MR results, in two separate tasks, to see how efficient this kind of handling of graphic results could be. Observers had no problems in separate congenital colour deficient subjects from normal trichromats using either test, reaching a 100% rate of success. In addition, observers faced no major problems in sorting out protans and deutans using the MR results. However, when using the FM 100 results they failed to agree about the classification of 10 out 91 congenital colour deficients without further discussion and quantification of the results. We concluded that both tests could be used for a quick screening of congenital colour deficient subjects, but some doubtful cases when using the FM 100 test should be thoroughly investigated with quantitative analysis of results. The FM 100 test has its limitations as it was shown in other works: it does not distinguish normal trichromats from mildly anomalous trichromats and does not always differentiate protans from deutans [39,40].

Possible discrepancies between the classification of protans and deutans obtained with the FM 100 test and the MR test were closely looked. There were five instances where the FM 100 test provided different results than the MR test. It was possible to study further two of these cases with anomaloscopy and molecular genetics and in both cases the results agreed with the MR results. We concluded that the MR test when available may provide a way to sort out doubtful results obtained with the FM 100 test. Part of the discrepancies faced with several investigators when using the FM 100 test, could arise from lightness differences in the printed colours used in the test; the changes in lightness are a clue to correct arrangement of the test without needing colour vision and this effect is accentuated in protans [2].

Some works compared results obtained with the CCT (commercial version of the MR test) and colour arrangement tests such as the D-15d [19]. Feitosa-Santana and colleagues studied type 2 diabetes with CCT and D-15d and concluded for the advantages of the CCT test compared to the D-15d test especially for its ability to detect and quantify a range of colour vision deficiencies [19].

Anomaloscopy performed with the Nagel Anomaloscope Mk 1 and the appropriate Rayleigh equation is considered to be the gold standard for the diagnosis of protan and deutan congenital colour vision deficiency [1,2]. The original Nagel Anomaloscope is not commercially available anymore but two new equipments are available, one of them (HMC anomaloscope) used to evaluate a subgroup of subjects studied in this work. Anomaloscopy was used in several studies where psychophysical determination of protan and deutan phenotypes using this technique was compared with the results obtained with other psychophysical tests [41,42] or molecular genetics determination of subject’s genotype [41,43,44]. Thus, ideally we would like to have been able to measure the Rayleigh equation of all subjects whose classification as protans or deutans differed in the two psychophysical tests, but that was not possible due to the availability of subjects for further testing. For the limited number of discrepant subjects (n = 3), agreement was obtained with the MR test.

Statistical analysis of the results obtained with the two tests gave quantitative support to the subjective analysis based on the visual inspection of graphic results. Congenital colour deficients differed significantly from normal trichromats in the number of mistakes they did in the FM 100 test and in the size and elongation of the five colour discrimination ellipses measured with the MR test. Statistical analysis was also used to efficiently sort out protans and deutans, providing significantly differences between these two groups for right central point in the FM 100 test and ellipse orientation in the MR test. We concluded that quantitative analysis of some parameters provided by FM 100 and MR tests was very efficient to separate congenital colour deficients from normal trichromats and protans from deutans.

It was also investigated which region of the colour space could be preferentially target to more efficiently sort out protans from deutans using the FM 100 test and the MR test. For the FM 100 test, this was done by performing the statistical analysis of the right and central points. Only the right central point location was a valuable parameter to separate protans from deutans, but this was done with some blur caused by the intrusion of some protans and deutans in the “wrong” region of the distribution of these two clusters of subjects (10 out 91 subjects). A more robust statistical analysis was performed comparing the orientations of colour discrimination ellipses in the colour space, once five different colour space locations were studied in the MR test. Ellipse #3 provided the largest difference between protans and deutans, Ellipse #1 provided little or no difference, and Ellipses #2, #4, and #5 were of intermediate value for this task. Multivariate analyses of all three parameters (size, elongation, and orientation) was also performed in the results from each of the five colour discrimination ellipses tested and, as expected, Ellipse #3 provided the best separation of protans and deutans in such analyses. We concluded that for a quick study of different group of congenital colour deficients, MR Ellipse #3 was the best choice.

These results could be explained by the colorimetric design of each test and its relationship to the characteristics of protan and deutan visions [2,45,46]. There are locations in the colour space where protan and deutan confusion axes are oriented relatively parallel to each other or defining low angular values at their intercept—in the CIE 1976 colour space these locations correspond to high values of *v’* (*v’* ≥ 0.5) [2]. There are other locations where protan and deutan confusion axes intercept at high angular values—in the CIE 1976 colour space these locations correspond to low values of *v’* (*v’* ≤ 0.42). In other locations, protan and deutan confusion axes intercept at intermediate angular values. Consequently, MR Ellipse #1 for protans and deutans have similar orientations, while MR Ellipses #3 for protans and deutans have orientations with the highest angular differences among all the five ellipses studied. A similar explanation could be given to the efficiency of the right and left central points to sort out protans and deutans [2].

## Conclusions

No one colour vision test may provide all the answer that a variety of needs of a service of optometry or ophthalmology. Thus, an association of different tests and sometimes the use of battery of tests may be recommended. In this work, the MR test was more sensitive than the FM 100 test, separated individuals by dysfunctional groups with greater precision and provided a more sophisticated quantitative analysis. In addition, the combination of FM 100 and MR tests might be appropriate for a more refined evaluation of different phenotypes of red-green colour vision deficiencies both congenital and acquired.
